# Cerebrospinal Fluid Aβ40 Improves the Interpretation of Aβ42 Concentration for Diagnosing Alzheimer’s Disease

**DOI:** 10.3389/fneur.2015.00247

**Published:** 2015-11-27

**Authors:** Aline Dorey, Armand Perret-Liaudet, Yannick Tholance, Anthony Fourier, Isabelle Quadrio

**Affiliations:** ^1^Center for Memory Resources and Research, Hospices Civils de Lyon, Charpennes Hospital, Lyon 1 University, Villeurbanne, France; ^2^Neurochemistry Unit, Biochemistry Department, Hospices Civils de Lyon, Groupement Hospitalier Est, Bron, France; ^3^BioRaN Team, Lyon Neuroscience Research Center, CNRS UMR 5292, INSERM U1028, Lyon 1 University, Bron, France; ^4^WAKE Team, Lyon Neuroscience Research Center, CNRS UMR5292, INSERM U1028, Lyon 1 University, Lyon, France

**Keywords:** dementia, Alzheimer, A**β**42, A**β**40, cerebrospinal fluid

## Abstract

The combination of decreased amyloid β42 (Aβ42) and increased total tau proteins (T-Tau) and phosphorylated tau (P-Tau) in cerebrospinal fluid (CSF) has recently been considered as a biological diagnostic criterion of Alzheimer’s disease (AD). Previous studies showed significant heterogeneity in CSF Aβ42 levels to discriminate AD from non-AD patients. It was also suggested that the CSF amyloid peptide β42/β40 ratio has better diagnostic performance than Aβ42 alone. The objective of the present study was to investigate the potential added value of determining CSF amyloid β40 peptide (Aβ40) for biological diagnosis of AD when CSF Aβ42 levels failed. CSF AD biomarkers were run in 2,171 samples from 1,499 AD and 672 non-AD patients. The following pathologic thresholds were used to define an AD-positive CSF biomarker profile: T-Tau ≥ 400 ng/L, P-Tau181 ≥ 60 ng/L, and Aβ42 ≤ 700 ng/L. CSF Aβ40 was assayed in AD patients with CSF Aβ42 levels above 700 ng/L and non-AD patients with CSF Aβ42 levels below 700 ng/L. CSF Aβ40 levels were higher in AD than non-AD patients. The receiver operator characteristic curves of CSF Aβ40 and the Aβ42/Aβ40 ratio defined AD cut-off values at 12,644 ng/L and 0.06, respectively. In AD patients with non-pathological CSF Aβ42, CSF Aβ40 concentration was able to correct 76.2% of cases when expressed as CSF Aβ42/Aβ40 ratio and 94.7% of cases when used alone. Using CSF Aβ42 and then CSF Aβ40, the percentage of misinterpreted AD patients fell to 1.0%. CSF Aβ40 concentration improved interpretation of Aβ42 level for the diagnosis of AD. CSF Aβ40 alone showed better diagnostic performance than the amyloid peptide Aβ42/Aβ40 ratio. The added value of determining CSF Aβ40 in AD diagnosis now needs confirming in a cohort of definite AD patients and to be completed with novel amyloid cascade biomarkers.

## Introduction

According to the revised criteria for Alzheimer’s disease (AD), definite diagnosis is founded on neuropathology as gold standard, when patients meet the clinical and cognitive criteria for AD dementia (1). Diagnosis of AD onset during the patient’s lifetime is said to be “possible” or “probable.” Amyloid β42 (Aβ42), total Tau (T-Tau), and phosphorylated Tau proteins (P-Tau) assay in cerebrospinal fluid (CSF) is recommended to increase the level of diagnostic certainty for AD in atypical clinical phenotypes, for inclusion of patients in clinical trials and to improve AD diagnosis at the earliest stages of the disease (1–5). A positive AD CSF biomarker profile was defined as increased CSF Tau and/or P-Tau181 and decreased CSF Aβ42 concentrations (1, 6–8). However, researchers and clinicians continue to debate the sensitivity and specificity of various biomarkers, and especially CSF Aβ42. A recent meta-analysis highlighted significant heterogeneity in CSF Aβ42 values between different disease groups (9), reporting sensitivity and specificity ranging from 71 to 91% and 44 to 82%, respectively. Moreover, Rosen et al. showed that “normal” CSF Aβ42 levels were observed in AD patients, leading to misinterpretation of the AD CSF biomarker profile in 23.2% of AD patients (10).

One of the crucial challenges to improve screening in clinical trials is to identify an accurate CSF biomarker reflecting amyloid pathology. There is now strong evidence that CSF Aβ42 levels depend not only on impaired brain clearance in Alzheimer’s pathophysiology, but also on the total load of amyloid peptides, which shows large interindividual variability (11–14). Gamma-secretase cleaves amyloid precursor protein (APP) at several sites, resulting in different C-terminally truncated Aβ variants: amyloid β40 (Aβ40) is the most abundant amyloid peptide in CSF (15), while Aβ42 accounts for only about 10% of the total Aβ peptide population (12, 16–18). Total Aβ concentration was found not to vary significantly between various dementia disorders (11, 18, 19), and Aβ40 concentration did not differ between AD (or presymptomatic AD) patients, healthy controls, and non-AD dementia patients (19–23). CSF Aβ40 concentration could, therefore, be considered to most closely reflect total Aβ load in the brain (13). Previous studies showed that the Aβ42/Aβ40 ratio in CSF is reduced in AD patients, and its assessment improves AD diagnostic accuracy (21–25). More recently, a few studies demonstrated added value for CSF Aβ40 or CSF Aβ42/Aβ40 ratio for differential diagnosis of AD using CSF P-Tau181 levels or in ambiguous AD CSF biomarker profiles (26–28). Therefore, the objective of the present study was to investigate whether determining CSF Aβ40 level and CSF Aβ42/Aβ40 ratio could improve diagnosis in AD patients without low CSF Aβ42 levels.

## Materials and Methods

Cerebrospinal fluid samples were collected between October 2010 and January 2013 from 2,171 patients who underwent lumbar puncture (LP) for routine clinical diagnosis of AD in the Neurochemistry Unit and Biochemistry Department of the University Hospital of Lyon (France). Patients were included in a multicenter memory clinic and had at least 2 years’ follow-up. They were classified into two groups: 1,499 AD and 672 non-AD patients. The non-AD group consisted of 259 patients with probable frontotemporal lobar degeneration (FTLD), 119 with probable dementia with Lewy bodies (DLB), 159 with normal pressure hydrocephalus (NPH), and 135 with psychiatric disorders.

The patients’ age, gender, and mini mental state evaluation (MMSE) score were recorded when the LP was performed. At that time, initial diagnosis was based on medical history, caregiver interviews, neurologic examination, neuropsychological battery evaluation, and brain imaging. Clinical diagnosis was made in multidisciplinary team meeting, comprising neurologists, neuropsychologists, and radiologists, and confirmed on follow-up. Dementia was defined according to DSM IV-TR criteria (29), and all AD patients were classified as having AD dementia with evidence of the AD pathophysiological process (1). Patients with mild cognitive impairment were excluded. The non-AD patients diagnosed with FTLD and DLB met the international criteria (30, 31). The non-AD patients with psychiatric disorders or NPH with cognitive complaints unrelated to AD or other degenerative disease were age matched with AD patients, and showed no progression of cognitive impairment within 2 years after CSF analysis.

This study, based on routine biological analyses, was not considered as “biomedical research” under French regulations, and therefore did not require informed consent. Samples were, however, stored in a biobank with authorization from the French Ministry of Health (Declaration number DC-2008-304). Authorization for handling personal data was granted by the French data protection commission [*Commission Nationale de l’Informatique et des Libertés* (CNIL)].

All patients underwent LP to collect CSF using a standard procedure. CSF collection, sampling, and storage were performed according to the international consensus (32, 33). All CSF samples were collected in Sarstedt polypropylene tubes (ref. 62.610.201) showing low adsorption of amyloid peptides (7). CSF biomarker analyses were performed, blind to clinical diagnosis, in the Neurochemistry Unit and Biochemistry Department of the University Hospital of Lyon. This department is involved in two external quality control schemes, one at French national level (working group of the French Society of Clinical Biology: *Société Française de Biologie Clinique*) and the other with the Alzheimer’s Association QC program (34). CSF concentrations of Aβ42, T-Tau, and P-Tau181 were measured using the standardized commercially available sandwich ELISA kit (INNOTEST^®^) according to the manufacturer’s procedures (Fujirebio, Ghent, Belgium).

For each CSF sample, Aβ42, T-Tau, and P-Tau181 biomarkers were simultaneously analyzed. As previously described (7), the cut-off values defining positive AD CSF biomarker profile were: T-Tau ≥ 400 ng/L, P-Tau181 ≥ 60 ng/L, and Aβ42 ≤ 700 ng/L.

Aβ40 level in CSF was quantified using ELISA tests [Human Amyloid b (1–40) (N) Assay kit, IBL, Japan] in AD patients with CSF Aβ42 levels above 700 ng/L and in non-AD patients with CSF Aβ42 levels below 700 ng/L.

### Statistical Analysis

Chi-square test, Mann–Whitney *U* test, Kruskal–Wallis test, and receiver operator characteristic (ROC) analyses were performed using MedCalc version 11.3.1.0 (http://www.medcalc.be). Differences were considered statistically significant at *p* < 0.05. ROC curves were applied to define optimal biomarker cut-off values to discriminate between AD and non-AD groups. The cut-off value was defined as the value corresponding to the highest average for sensitivity and specificity. Accuracy was calculated as the sum of true positives and true negatives in the total number of patients (35).

## Results

**Figure 1:**
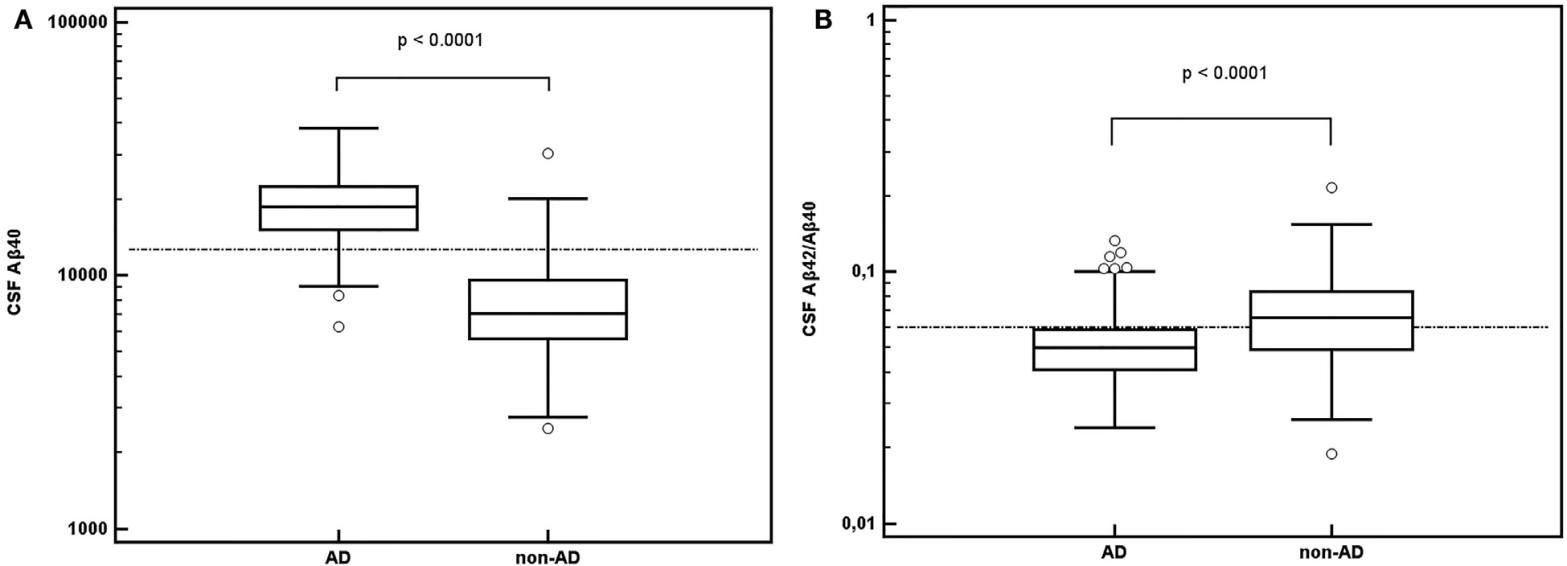
**CSF A**β**42/A**β**40 ratio (A) and CSF A**β**40 concentrations in nanograms per liter (B) in AD and non-AD populations**. Abbreviation: AD: Alzheimer’s disease.

Cerebrospinal fluid data according to diagnostic group are summarized in Table 1 and Figure 1.

**Table 1:** **Demographic, pathologic, and biological parameters of study populations**.

		AD	Non-AD
Gender	*n*	1,499	672
	M/F	643/856	358/314
Age (years)	*n*	1,499	672
	Mean	71.6	70.0
	SD	9.5	10.6
MMSE score (/30)	*n*	1,093	488
	Mean	20.2	21.6
	SD	5.6	5.5
T-Tau (ng/L)	*n*	1,499	672
	Median	650	230
	25th–75th P	487–913	168–311
P-Tau_181_ (ng/L)	*n*	1,499	672
	Median	83	38
	25th–75th P	68–109	30–48
Aβ42 (ng/L)	*n*	1,499	672
	Median	539	807
	25th–75th P	443–663	570–1,056
Aβ40 (ng/L)	*n*	281	244
	Median	19,198	7,112
	25th–75th P	15,162–22,409	5,643–9,636
Aβ42/Aβ40 ratio	*n*	281	244
	Median	0.053	0.066
	25th–75th P	0.041–0.059	0.049–0.084

*AD, Alzheimer’s disease; MMSE, mini mental state evaluation; M, male; F, female; SD, standard deviation; P, percentile*.

**Figure 2:**
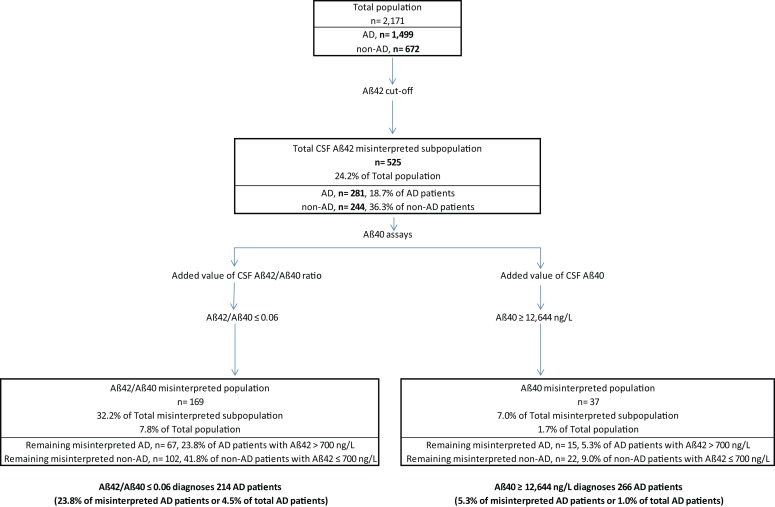
**Patient classification based on the determination of cerebrospinal fluid (CSF) amyloid peptides**. First, according to CSF Aβ42 levels, we obtained a percentage of misinterpreted patients with discordant results regarding clinical diagnosis. The CSF Aβ40 assay was performed in this subpopulation. Performance in accurately classifying patients was tested for CSF Aβ42/Aβ40 ratio and for CSF Aβ40 alone. Both CSF Aβ42/Aβ40 ratio and CSF Aβ40 could reclassify a high percentage of patients. CSF Aβ40 provided the best correct classification rate. Abbreviation: AD: Alzheimer’s disease.

About 81.3% of AD patients (1,218/1,499) fulfilled the pathological CSF Aβ42 criteria; the remaining 18.7% (281/1,499) presented CSF Aβ42 levels above cut-off (>700 ng/L). 63.7% of non-AD patients (428/672) presented CSF Aβ42 levels above 700 ng/L; 36.3% (244/672) had CSF Aβ42 levels below 700 ng/L (Figure 2). CSF Aβ40 levels were then determined in these 525 patients: 281 AD patients (>700 ng/L) and 244 non-AD patients (≤700 ng/L).

**Figure 3:**
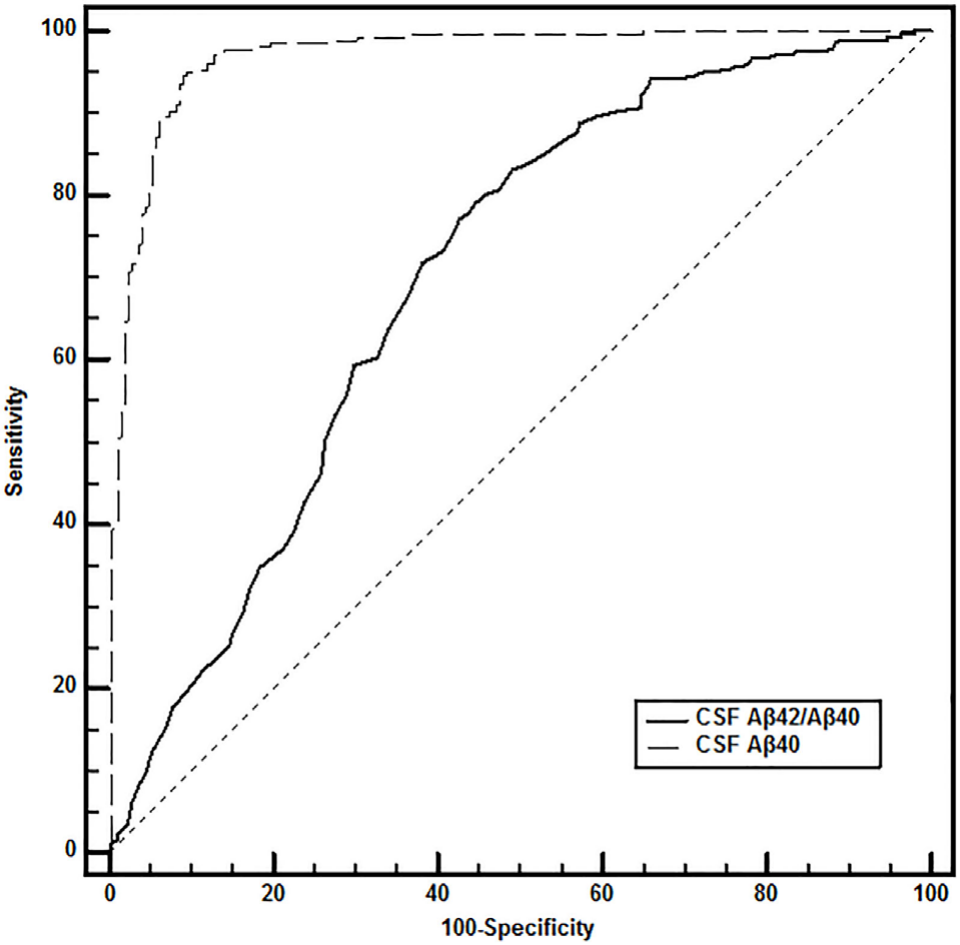
**Receiver operating characteristic curve comparison for AD diagnosis in the “discordant CSF A**β**42 values” subpopulation**. DeLong et al.’s (1988) method was used to compare the values of the area under the curve (AUC). In the 525 selected patients, accuracy of diagnostic performance was significantly higher for CSF Aβ40 compared to CSF Aβ42/Aβ40 ratio, with 94.7% sensitivity and 91.0% specificity for CSF Aβ40 ≥12,644 ng/L (AUC, 0.969) compared to 76.2 and 58.2%, respectively for CSF Aβ42/Aβ40 ratio ≤0.06 (AUC, 0.700).

The ROC curves of CSF Aβ40 level and the Aβ42/Aβ40 ratio determined AD cut-off values of ≥12,644 ng/L and ≤0.06, respectively (Figure 3).

In the overall population, the percentage of patients in whom amyloid pathology was misinterpreted fell from 24.2% (525/2,171) using CSF Aβ42 alone to 7.8% (169/2,171) when it was followed by CSF Aβ42/Aβ40 ratio, and to 1.7% (37/2,171) when followed by CSF Aβ40 (Figure 2). In patients in whom CSF Aβ40 level was determined (*n* = 525), sensitivity and specificity for AD diagnosis were 76.2 and 58.2%, respectively (accuracy, 0.678) using the CSF Aβ42/Aβ40 ratio, and 94.7 and 91.0%, respectively (accuracy, 0.930) using CSF Aβ40 determination.

About 58.2% of the 244 non-AD patients with CSF Aβ42 levels below 700 ng/L (142/244) had CSF Aβ42/Aβ40 ratios higher than 0.06 and 91.0% (222/244) had CSF Aβ40 levels below 12,644 ng/L.

About 76.2% of AD patients (214/281) had CSF Aβ42/Aβ40 ratios below 0.06 and 94.7% (266/281) had CSF Aβ40 levels higher than 12,644 ng/L. In the overall AD population, percentage misinterpretation fell from 18.7% (281/1,499) with CSF Aβ42 alone to 4.5% (67/1,499) using CSF Aβ42 and then CSF Aβ42/Aβ40 ratio and 1.0% (15/1,499) using CSF Aβ42 and then CSF Aβ40 (Figure 2).

## Discussion

We investigated the potential added value of CSF Aβ40 assay to improve the interpretation of Aβ42 level. The main finding was that CSF Aβ40 appeared to be an interesting complementary biomarker. CSF Aβ40 levels were higher in AD than non-AD patients. Thus, determining CSF Aβ40 concentrations corrected biological diagnosis in AD patients with non-pathological CSF Aβ42 levels in 76.2% of cases using the CSF Aβ42/Aβ40 ratio and in 94.7% using CSF Aβ40 alone; using CSF Aβ42 and then CSF Aβ40, percentage misinterpretation fell to 1.0%.

Cerebrospinal fluid Aβ42 concentrations led to misinterpretation of the AD CSF biomarker profile in 24.2% of our total population and notably in 18.7% of AD patients. This low performance of CSF Aβ42 is in perfect agreement with previous reports (7, 10, 18, 20, 36, 37). The presence of CSF Aβ42 concentrations ≤700 ng/L in non-AD patients could reflect low total CSF amyloid load, while CSF Aβ42 >700 ng/L in AD patients could result from high amyloid load. This concept justifies CSF Aβ40 assay to complete amyloid pathway interpretation.

As reported in various studies (20, 26, 27, 36), the CSF Aβ42/Aβ40 ratio showed better diagnostic performance than CSF Aβ42 alone. The CSF Aβ42/Aβ40 ratio cut-off value at 0.06 was identical to that reported by Lewczuk et al. (36). The discrepancy with Hansson et al.’s (20) 0.095 cut-off might be due to the Genetics Company ELISA kit halving the range of CSF Aβ40 levels. We found an increase in the rate of correct interpretation from 75.8% with CSF Aβ42 alone to 92.2% when CSF Aβ42 assay was followed by determining the CSF Aβ42/Aβ40 ratio, similarly to other reports (20, 28, 36).

The type of sampling and storage tubes is an important source of variability because of amyloid adsorption (33, 37, 38). CSF sample selection from biological banks should, therefore, be performed rigorously. There is parallel adsorption of CSF Aβ42 and Aβ40 onto the sampling tube surface, regardless of the type of plastic (personal data). Systematic use of the CSF Aβ42/Aβ40 ratio would provide complete interpretation of CSF amyloid biomarker results, integrating the impact of plastic tube type. In the present study, however, samples were analyzed sequentially, leading to higher between-run imprecision for the CSF Aβ42/Aβ40 ratio than for CSF Aβ42 alone [coefficient of variation (CV), 13.3 and 10.2%, respectively]. One solution to decrease the CV of the CSF Aβ42/Aβ40 ratio would be to use multiplex assays to analyze both amyloid peptides simultaneously. Unfortunately, at the moment, there is no analytical validation available for CSF Aβ42 and CSF Aβ40 in multiplex assays for *in vitro* diagnostic use.

In the present study, CSF Aβ40 was determined only in AD patients with CSF Aβ42 levels above 700 ng/L and in non-AD patients with levels below 700 ng/L. CSF Aβ40 concentrations were significantly higher in AD than non-AD patients. The optimal CSF Aβ40 cut-off value was 12,644 ng/L. To our knowledge, there is currently no effective CSF Aβ40 cut-off value to discriminate AD from non-AD patients reported in the literature; only a slight increase in CSF Aβ40 was found in two other studies (20, 24), and a recent study focusing on AD-MCI patients found a significant increase in CSF Aβ40 values compared to a control group (36). However, the present data contrasted with those reported in another study (26) including AD and non-AD dementia. Selection of the non-AD patient population to compare with the AD population was probably one of the major differences. Another difference may be the biological factor used for the patients’ initial classification, CSF P-Tau181 concentrations in intermediate levels (26). Similarly, Sauvee et al. suggested using the CSF Aβ42/Aβ40 ratio when data for CSF Aβ42 combined to CSF P-Tau181 are inconclusive (27). In these particular cases, adding the CSF Aβ42/Aβ40 ratio improved their proportion of interpretable biological profiles from 68 to 89% (27). Moreover, in confirmation of our sequential approach, Sauvee et al. showed that adding CSF Aβ40 peptide concentration and CSF Aβ42/Aβ40 ratio did not change their conclusions when CSF Aβ42 and CSF P-Tau181 were concordant.

In the present study, it was also interesting that 36.3% of non-AD patients presented pathological CSF Aβ42 levels. One hypothesis could concern the heterogeneity of the non-AD population, which included patients with psychiatric disorders and NPH and demented patients with neurodegenerative diseases (FTLD and DLB). CSF Aβ42 was previously reported to be less effective for differential diagnosis of the main neurodegenerative dementia than CSF Tau proteins (39–41). To discriminate AD and FTLD, CSF Aβ42 assay could then be combined with Tau proteins and expressed as T-Tau/Aβ42 and P-Tau181/Aβ42 ratios (42, 43). Typical CSF AD profiles including CSF Aβ42 and Tau proteins were reported in 47% of patients meeting clinical diagnostic criteria for DLB and in 30% of FTLD patients (41), suggesting coexisting pathologies, as strongly highlighted by postmortem studies (44, 45). NPH patients also have lower CSF amyloid peptide and Tau protein concentrations than controls (46, 47). To validate our hypothesis and strategy regarding differential diagnosis, postmortem confirmation on autopsy-proven patients should be carried out.

The diagnostic performance of CSF Aβ42 is increasingly questioned. It should be noted that biological diagnosis as performed in specialized memory clinics is also founded on the second pathway of AD pathophysiology, reflected by CSF Tau protein levels. Nevertheless, a more accurate evaluation of CSF amyloid biomarkers is important to include patients in therapeutic trials involving the amyloid cascade, using added Aβ peptides or other amyloid cascade biomarkers. For example, the soluble peptide APPβ (sAPPβ) and CSF Aβ40 come from the same enzymatic digestion of APP, and it would be interesting to assess sAPPβ to complete this study. Increased CSF sAPPβ levels were already reported in AD patients as compared to non-AD demented patients (48) and FTD patients (49).

In conclusion, the present study offers an improvement in biological diagnosis of AD focusing on the amyloid pathway. In the misinterpretation using CSF Aβ42 levels, classification based on the CSF Aβ42/Aβ40 ratio gives good results. More interestingly, CSF Aβ40 assay alone also provides better results: the misinterpretation rate using CSF Aβ42 and then CSF Aβ40 alone falls to 1.7%. Sequential assessment of CSF Aβ40 would also provide a better cost-effectiveness ratio than systematic determination of the CSF Aβ42/Aβ40 ratio. Finally, these results need to be confirmed in a prospective study including autopsy-proven AD patients, and completed with novel amyloid cascade biomarkers.

## Conflict of Interest Statement

The authors declare that the research was conducted in the absence of any commercial or financial relationships that could be construed as a potential conflict of interest.
